# The Diversity of the Pollen Tube Pathway in Plants: Toward an Increasing Control by the Sporophyte

**DOI:** 10.3389/fpls.2016.00107

**Published:** 2016-02-09

**Authors:** Jorge Lora, José I. Hormaza, María Herrero

**Affiliations:** ^1^Department of Subtropical Fruit Crops, Instituto de Hortofruticultura Subtropical y Mediterránea La Mayora – University of Málaga – Consejo Superior de Investigaciones CientíficasMálaga, Spain; ^2^Department of Pomology, Estación Experimental Aula Dei, Consejo Superior de Investigaciones CientíficasZaragoza, Spain

**Keywords:** pollen tube, sporophyte, gametophyte, pistil, evolution

## Abstract

Plants, unlike animals, alternate multicellular diploid, and haploid generations in their life cycle. While this is widespread all along the plant kingdom, the size and autonomy of the diploid sporophyte and the haploid gametophyte generations vary along evolution. Vascular plants show an evolutionary trend toward a reduction of the gametophyte, reflected both in size and lifespan, together with an increasing dependence from the sporophyte. This has resulted in an overlooking of the importance of the gametophytic phase in the evolution of higher plants. This reliance on the sporophyte is most notorious along the pollen tube journey, where the male gametophytes have to travel a long way inside the sporophyte to reach the female gametophyte. Along evolution, there is a change in the scenery of the pollen tube pathway that favors pollen competition and selection. This trend, toward apparently making complicated what could be simple, appears to be related to an increasing control of the sporophyte over the gametophyte with implications for understanding plant evolution.

## Introduction

Alternation of generations between gamete-producing multicellular gametophytes and spore-producing sporophytes is present in all land plants (Hofmeister, 1851). However, along the evolutionary line, the gametophytic phase gets reduced in terms of both size and lifespan compared to the sporophytic phase (Heslop-Harrison, 1975). In bryophytes the gametophytic generation is the more prominent phase and the sporophyte is nutritionally dependent on the gametophyte (Maciel-Silva and Porto, 2014). However, this situation is reversed in seed plants, in which the sporophyte is the prominent phase and the gametophytic generation is the one that develops and spends most of its life enclosed within the tissues of the sporophyte. Due to this prevalence of the sporophytic phase, often the implications of the gametophytic phase in plant evolution and domestication have been overlooked.

The mature pollen is the haploid microgametophyte. Mature pollen of gymnosperms is highly variable and can be formed by up to forty cells (Sterling, 1963; Pacini et al., 1999). However, a trend toward decreasing the number of cells could have appeared early in gymnosperm evolution (Sterling, 1963), probably linked to an evolutionary advantage related to a more efficient energy use (Pacini et al., 1999). In angiosperms, the mature male gametophyte is reduced to three cells, the vegetative cell and the two sperm cells. The female gametophyte (embryo sac) is generally formed by seven cells (two synergid cells, the egg cell, the central cell and three antipodal cells) embedded in the sporophytic tissues of the ovule. Interestingly, in spite of the wide morphological diversity observed for most traits and structures in the sporophytic phase of flowering plants, the development, structure, and function of the male and female gametophytes are very well conserved across angiosperm lineages.

However, clear differences in the subsequent contribution of the sporophytic tissues that envelop the female gametophyte and interact with the male gametophyte are observed along evolution. This is shown in the male–female meeting place where pollen germinates, and also along the pollen tube pathway toward the female gametophyte. All these steps have been widely studied in seed plants, but more extensively in angiosperms (Whitehead, 1969; Mulcahy, 1979; Eriksson and Bremer, 1992; Herrero, 1992; Hormaza and Herrero, 1992; Sargent and Otto, 2004), than in gymnosperms (Friedman, 1993, 2015; Owens et al., 1998; Gelbart and von Aderkas, 2002). In angiosperms, pollen germination and pollen tube growth takes place within the carpel that has been suggested to provide an opportunity for pollen competition and selection (Mulcahy, 1979; Hormaza and Herrero, 1992; Herrero and Hormaza, 1996; Taylor and Kirchner, 1996; Erbar, 2003; Lankinen and Green, 2015). However, variations in carpel structure are apparent along the evolution. Here we examine these differences, paying especial attention to the interaction between the male gametophyte and the female sporophyte during the different steps of the pollen tube pathway. We first examine the conservation of a pollen tube and then follow the pollen tube pathway, where a change in the meeting place and variations in the style and the ovary are observed. Finally, the implications of these variations during plant evolution are considered to get an integrated view on the evolutionary trends in the control of pollen tube performance in seed plants and the role of the sporophyte in this process.

## The Conservation of a Pollen Tube

**FIGURE 1:**
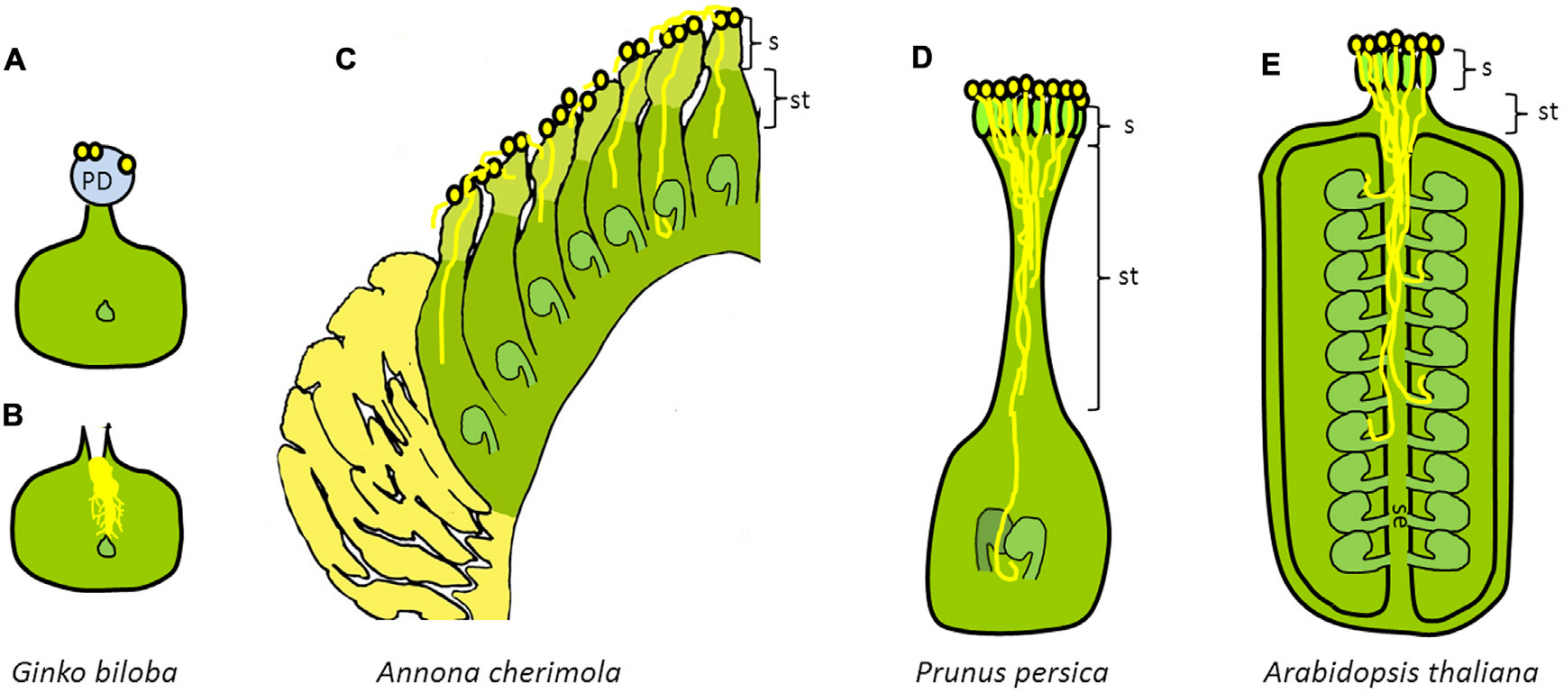
**Diversity of pollen tube pathways in seed plants.** Schematic cross section of the gymnosperm carpel of **(A,B)** *Ginkgo biloba*, modified from Friedman (1987) and the angiosperm carpel of **(C)** *Annona cherimola*, **(D)** *Prunus persica*, and **(E)** *Arabidopsis thaliana*. **(A,B)** Gymnosperm carpel of **(A)** *Ginkgo biloba* showing the pollination drop and **(B)** pollination drop retraction with a branched pollen tube. **(C)** Angiosperm carpel of an early divergent angiosperm (*Annona cherimola*) showing extragynoecial compitum and unfused carpels (apocarpous). **(D)** Angiosperm carpel of an evolutionarily derived angiosperm (*Prunus persica*) showing pollen competition in the long style. **(E)** Angiosperm carpel of an evolutionarily derived angiosperm (*Arabidopsis thaliana*) showing pollen competition in the style and septum of a syncarpous gynoecium. PD, pollination drop; S, stigma; ST, style; SE, septum.

A comparative review of the evolution of the male gametophyte (Friedman, 1993; Rudall and Bateman, 2007) shows that the most basal groups of extant gymnosperms, cycads, and Ginkgoales (Chaw et al., 2000), have multiflagellate sperms that swim to reach the archegonia [zooidogamy] (Friedman, 1990) whereas conifers (Fernando et al., 2005), Gnetales (Chaw et al., 2000) and angiosperms release non-motile male gametes through siphonogamy (Friedman, 1993). In fact, since the first pollen tube was observed by Amici (1824) in the angiosperm *Portulaca oleracea* in the early 19th century, pollen tubes were consistently observed in very different seed plant species. Evidence of microgametophyte tubes have also been observed in other plant taxa such as paleozoic seed ferns (Rothwell, 1972), where the tube was found in a chamber showing a branching pattern that can also be found in gymnosperms (Little et al., 2014). Thus, pollen tube development of *Ginkgo biloba* shows an initial tubular multiaxial form (**Figure 1**) (Friedman, 1987), cycad male gametophytes typically show unbranched pollen tubes although they can also be slightly branched (Friedman, 1987), and conifers show pollen tubes that can vary from unbranched to extensively branched (Fernando et al., 2005). Branching in gymnosperms has been suggested to have a haustorial role (Sterling, 1963; Little et al., 2014). In this sense, the heterotrophic pollen tube growth of most gymnosperms is associated with the degeneration of the invaded cells of the sporophyte by cellular outgrowth or enzymatic action. Thus, in gymnosperms, nucellar cell degeneration produced by pollen tube growth has been observed in *Zamia furfuracea* (Cycadaceae) (Choi and Friedman, 1991) and in *Pseudotsuga menziesii* (Pinaceae) (Owens and Morris, 1990). In angiosperms, heterotrophic pollen tube growth at the expenses of the stylar reserves was early documented (Herrero and Dickinson, 1979). Pollen tube branching has also been reported in several angiosperm species (Wilms, 1974; Hill and Lord, 1987; Smith-Huerta, 1991; Johri, 1992; Sogo and Tobe, 2005), but no consensus on the cause or function of this branching has yet been reached. Non-mutually exclusive hypotheses include a haustorial role (Johri, 1992), a response to the clumping of pollen grains on the stigma (Smith-Huerta, 1991), interspecific (Williams et al., 1982), and intraspecific (Seavey and Bawa, 1986) incompatibility responses, or chalazogamy -the entrance of the pollen tube through the chalaza instead of the common way through the micropyle- (Sogo and Tobe, 2005, 2006b). But all these different situations may share a common ground: the absence of proper guidance signals for pollen tube growth from underdeveloped ovules (Herrero, 2000, 2001, 2003; Sogo and Tobe, 2006a).

Once the pollen tubes reach their target, fertilization takes place after the release of the male gametes from the pollen tube to the fertilization chamber in gymnosperms, or to the egg apparatus in angiosperms. Interestingly, morphological evidence suggests that the cytoskeleton of sperm cells and generative cells of angiosperms are closely related to the cytoskeleton of the flagellated sperm cells (Southworth and Cresti, 1997).

The Gnetales, with the exception of *Welwitschia* (Friedman, 2015), show some additional features that are also found in angiosperms. Thus, double fertilization occurs in *Ephedra* (Friedman, 1990; Friedman and Carmichael, 1996), and *Gnetum* displays a cell–cell interaction between the pollen tube and nucellar cells in an angiosperm-like fashion (Bell, 1995). Taken together this information, it appears clear that the construction of a pollen tube provides a prevalent useful way for the male gametes to travel toward the female gametophyte. But the landing site and the length of the pollen tubes in the different species are very variable, depending on the distance needed to travel to reach the targeted female gametophyte.

## Changing the Meeting Place

The first meeting point between the male gametophyte and the female sporophyte changes along the evolution of seed plants. Thus, in most gymnosperms, pollen lands in a pollination drop on the micropyle (**Figure 1**), which is reabsorbed with pollen and other air-borne particles (Gelbart and von Aderkas, 2002; Little et al., 2014). The situation is different in angiosperms in which a major synapomorphy is the presence of the ovule enclosed in a carpel (Endress and Igersheim, 2000). The carpel adds an additional envelope to the female gametophyte and also a different landing place for the pollen grain, the stigma (**Figure 1**).

In gymnosperms, the pollination drop is a viscous liquid secretion produced by the ovule, usually by the nucellar tissue (Gelbart and von Aderkas, 2002; Coulter et al., 2012), with the probable contribution of other tissues such as the megagametophyte and the integument (Owens et al., 1998). The pollination drop is composed of carbohydrates, amino acids, and a miscellany of other compounds (Gelbart and von Aderkas, 2002; Nepi et al., 2009; von Aderkas et al., 2015). Pollination drops are commonly found in Ginkgoales (Friedman, 1987), Cycadales (Choi and Friedman, 1991), Gnetales (Endress, 1996; Bolinder et al., 2015; Friedman, 2015; von Aderkas et al., 2015), and Pinales (Owens et al., 1998; Möller et al., 2000; Chandler and Owens, 2004), although no pollination drop seems to be produced in *Abies*, *Cedrus*, *Tsuga*, *Pseudotsuga*, or *Larix* in which pollen is drawn into the micropyle by collapse or inward growth of integument cells (Sedgley and Griffin, 1989). Captured pollen triggers a retraction of the drop that facilitates access of the pollen to the micropyle. While pollen from unrelated species can initiate the retraction of the pollination drop, the process is only completed by conspecific pollen (Mugnaini et al., 2007; Jin et al., 2012). Environmental conditions can significantly affect the success of pollination since the pollination drop can evaporate under low relative humidity conditions (Gelbart and von Aderkas, 2002). Conversely, the withdrawal of the pollination drop can be inhibited by high relative humidity (Mugnaini et al., 2007; Jin et al., 2012). In Pinaceae a pollination drop has not been identified and it has been proposed that rainwater could take its function (Owens et al., 1998; Leslie, 2010). Besides favoring or jeopardizing the production of the pollination drop, environmental factors may also provide clues for its production, as the situation recently reported in insect-pollinated *Ephedra foeminea* where the pollination drop is produced during the full moon of July (Rydin and Bolinder, 2015). Taken together, these observations suggest that the pollination drop, and hence a successful pollination in gymnosperms, is rather vulnerable to environmental conditions. In fact, in both angiosperms and gymnosperms, pollen capture is highly influenced by environmental factors (Heslop-Harrison and Heslop-Harrison, 1985; Heslop-Harrison, 2000); however, in angiosperms the situation changes, showing a higher involvement of the sporophyte in the process due to the presence of closed carpels.

In fact, in angiosperms pollen landing occurs in a highly specialized structure: the stigma. While several molecules such as surface esterases, arabinogalactan proteins (AGPs), low-esterified homogalaturonans, or glycoside hydrolases can be found in both stigma and pollination drops (Coulter et al., 2012) and could be analogous (Villar et al., 1984), the stigma represents a major evolutionary transition that provides adhesion, hydration, and germination media for the pollen grains (Sanzol et al., 2003; Hiscock, 2008). A number of factors, such as reactive oxygen species (ROS; McInnis et al., 2006; Allen et al., 2011; Traverso et al., 2013; Serrano et al., 2015) commonly proposed to mediate cell–cell communication (Gilroy et al., 2014), or surface esterases (Hiscock et al., 2002) contribute to this receptivity. In apple flowers, AGPs are secreted to the stigma concomitantly with the acquisition of stigmatic receptivity (Losada and Herrero, 2012) and their role supporting pollen germination is shown by the fact that they vanish following pollen germination. AGPs have been found in the stigma of several early divergent angiosperms (Prychid et al., 2011; Costa et al., 2013; Losada et al., 2014), and recent work shows that they also mark stigmatic receptivity in *Magnolia* (Losada et al., 2014), strongly suggesting that the secretion of AGPs could well be a conserved process in flowering plants marking stigmatic receptivity. Interestingly, AGPs were also reported in the pollination drop of the gymnosperm *Taxus x media* (Coulter et al., 2012). Low-esterified homogalacturonans are also involved in the adhesion of the pollen to the stigma of angiosperms (Mollet et al., 2000) and seem to be play a role also in pollen grain adhesion in the gymnosperm *Larix decidua* (Rafińska et al., 2014). Glycoside hydrolases that are required for cell wall elongation and, consequently, for pollen tube growth, are found in both the angiosperm stigmas and the gymnosperm pollination drop, but expansins that are also involved in cell wall elongation are only found in the stigma of angiosperms (Coulter et al., 2012).

Stigmatic receptivity usually provides a short window of opportunity for pollination to be effective (Sanzol et al., 2003). This window is influenced by environmental factors, basically temperature at flowering (Hedhly et al., 2003, 2005b; Lora et al., 2011a; Sage et al., 2015). High temperatures accelerate stigma degeneration, whereas low temperatures maintain the stigma receptive for a longer time, affecting the effective pollination period (Sanzol and Herrero, 2001).

Once the pollen grains have germinated in the ovular pollination drop in gymnosperms or in the stigma in angiosperms, the pollen tube grows to reach the female gametophyte. The pollen tube pathway takes place surrounded by female sporophytic tissues and, consequently, with less environmental dependence but higher male–female interaction. Moreover, in angiosperms, this journey is further complicated by the presence of a new structure, the style.

## The Style: Making a Short Story Long

In most angiosperms, from pollen landing on the stigma to fertilization inside the ovary, the pollen tube has to traverse an intermediate territory, the style (**Figure 1**). The pollen tube grows through a stylar canal or a stylar transmitting tissue, and reaches the locule cavity of the ovary where the ovule is located. In the basal grade of angiosperms and magnoliids the style is very short or even absent and the stigma forms the margins of the sealed but unfused carpel in a continuum with the placenta leading to the ovule (Endress, 2015). Examples of these observations include basal angiosperms, such as *Amborella trichopoda* (Williams, 2009), *Nymphaea capensis* (Orban and Bouharmont, 1995), or *Cabomba caroliniana* (Galati et al., 2016), and magnoliids such as *Annona cherimola* (Lora et al., 2010, 2011a), *Magnolia virginiana* (Losada et al., 2014), or *Saururus cernuus* (Pontieri and Sage, 1999).

The stylar canal, or the stylar transmitting tissue, provides an extracellular secretion that is incorporated into the growing pollen tubes and allows a heterotrophic pollen tube growth (Labarca and Loewus, 1973; Herrero and Dickinson, 1979), that differs from the autotrophous pollen tube growth in the stigma (Herrero and Dickinson, 1981; Stephenson et al., 2003). This role of the pistil providing nutrients for the growing pollen tubes has been shown in a wide range of angiosperms including many fruit tree crops (Herrero and Arbeloa, 1989; Gonzalez et al., 1996; Martinez-Palle and Herrero, 1998; Alcaraz et al., 2010; Distefano et al., 2011; Suárez et al., 2013), and seems to be a conserved process in flowering plants. Secretion along the short style is also already present in basal angiosperms [*Trimenia moorei*, *Illicium floridanum* (Bernhardt et al., 2003), *Kadsura longipedunculata* (Lyew et al., 2007), or *Amborella trichopoda* (Thien et al., 2003)] as well as in early divergent angiosperms [*Saururus cernuus* (Pontieri and Sage, 1999), *Psedowintera axillaris* (Sage and Sampson, 2003), or *Magnolia virginiana* (Losada et al., 2014)]. The implication of different molecules in pollen–pistil interaction has been put forward with a role in providing adhesion and facilitating pollen tube growth through the transmitting tissue (Boavida et al., 2011; Chae and Lord, 2011; Palanivelu and Tsukamoto, 2012; Qu et al., 2015).

A number of different molecules that contribute to pollen tube building and guidance have been found in the style of angiosperms and a similar scenario has also been proposed in the gymnosperm *Larix decidua* (Rafińska et al., 2014). Concomitantly with pollen tube growth, an increase in the amount of calcium in the extracellular matrix has been observed (Lenartowska et al., 2001; Ge et al., 2009), which suggests the provision of an optimal calcium environment for polar pollen tube growth (Hepler et al., 2012; Steinhorst and Kudla, 2013). The role of calcium on pollen grain germination and pollen tube growth was also shown in conifers (Chen et al., 2008; Lazzaro et al., 2013). Suggesting complex needs for both gymnosperm and angiosperm pollen germination and tube growth. Other signals from the stigma and style, such as stigma/style cysteine-rich adhesins (SCA) or chemocyanins, have also been shown in lily (Chae and Lord, 2011). While SCA are involved in pollen tube tip growth (Chae and Lord, 2011; Qu et al., 2015), chemocyanins appear to play a role in adhesion of the pollen tube to the stylar tissue (Park et al., 2000). Plantocyanins, blue copper proteins expressed also in the stylar transmitting tissue and homologs of chemocyanins, are also involved in pollen tube guidance, which is disrupted by plantocyanin overexpression in *Arabidopsis* (Dong et al., 2005). Experiments performed in maize also showed the sporophytic control of pollen tube guidance through the transmitting tissue that it is unaffected in RNAi-lines lacking functional embryo sacs (Lausser et al., 2010).

Recent studies have revealed the role of AGPs on pollen tube growth (Pereira et al., 2015) that have also been detected in the pollen tube tip of angiosperms (Qin et al., 2007; Dardelle et al., 2010). AGPs were observed in the stigma at pollination time in *Magnolia* (Losada et al., 2014), where a proper style is missing, and in olive (*Olea europaea*) (Suárez et al., 2013) that has a reminiscent style. Interestingly, in apple flowers which have a proper long style, AGPs were clearly present in the stigma but were not observed in the stylar transmitting tissue. Conversely, extensins that are involved in cell wall extension could not be detected in the stigma, but filled the style (Losada and Herrero, 2014). The clear boundary in two adjacent territories (stigma and style) of angiosperms, in which pollen tube kinetics are clearly different, suggests a support for faster pollen tube growth in the style. The remaining question is the possible meaning of this accelerated pollen tube growth in the style and the biological significance of the acquisition of a long style that requires the building up of a long pollen tube, making long, and complicated what could be a short and simple process.

A rapid pollen tube growth rate has been considered a key innovation of angiosperms (Williams, 2008). Thus, the whole process from pollination to fertilization can take several months in gymnosperms, which generally show slower pollen tube growth rates than angiosperms. An exception could be *Ephedra*, where a rapid fertilization has been reported (Land, 1907), but associated with a short pollen tube pathway and, consequently, a slow pollen tube rate similar to that observed in other gymnosperms (Williams, 2008). Rapid fertilization associated with a fast pollen tube growth rate was observed in the extant ANITA grade and magnoliids where pollen tubes penetrated the ovule in some 9–48 h after pollination (Bernhardt et al., 2003; Williams, 2008, 2012a,b; Lora et al., 2010).

However, in addition to the trend toward a faster pollen tube growth rate observed in seed plants, the process is also highly dependent on temperature (Hedhly et al., 2004; Coast et al., 2016). Thus, pollen tube growth is often slower in species adapted to temperate climates; for example, the progamic phase from pollination to fertilization takes three weeks in *Prunus persica* (Herrero and Arbeloa, 1989). Still, temperature cannot be the only factor influencing pollen tube growth rate since species flowering under similar temperatures have clear differences in the length of the progamic phase, as it occurs between peaches and apricots (Herrero and Arbeloa, 1989; Rodrigo et al., 2000). Most significantly, a slow pollen tube growth rate appears to be related to a delayed maturation of the pistil in the ovary (Herrero and Arbeloa, 1989).

## The Ovary: A Hidden Intense Interaction Area

In both gymnosperms and angiosperms the seeds that will produce the next sporophytic generation develop from the ovules. In angiosperms, once the pollen tubes get to the base of the style, they face the ovary that encloses the ovule(s). Angiospermy, or the presence of a closed carpel, is considered a key innovation of angiosperms (Endress and Igersheim, 2000; Endress, 2015), although variations in the degree of closure of the carpel have been described (Endress and Igersheim, 2000), ranging from sealing by secretion without postgenital fusion to complete postgenital fusion with intermediate stages. In the ANITA grade, carpel sealing is usually without postgenital fusion (Endress and Igersheim, 2000). In the magnoliids *Annona cherimola* (Lora et al., 2010) and *Magnolia virginiana* (Losada et al., 2014), papillae along the suture line face each other in a zip like fashion, with secretion filling the gaps and the pollen tubes grow along this continuous zip that goes from the stigma down to the ovule. In more derived clades, the process is more complex and the pollen tube has to enter the ovary, where particular structures may protect this entrance (Herrero, 2000). In peach flowers, the obturator -a placental protuberance facing the ovule- regulates this access, since pollen tube growth is only possible when this structure enters a secretory phase (Arbeloa and Herrero, 1987). The obturator has also been described in basal angiosperms such as members of the Austrobaileyales (*Kadsura longipedunculata*) (Lyew et al., 2007), and early divergent angiosperms in the magnoliid clade [*Annona cherimola* (Lora et al., 2010) and *Persea americana* (Sedgley and Annells, 1981)], although its active role in promoting pollen tube passage was not observed. This may be related to a continuous secretion, as it occurs in *Annona cherimola* where secretion is continuous all along the suture line right from anthesis up to fertilization time (Lora et al., 2010). A similar situation has been recorded in kiwifruit flowers with obturators secreting right from anthesis up to fertilization time (Gonzalez et al., 1996), which occurred some 3 days after pollination. Conversely, in peach flowers, this process was much slower lasting for about three weeks (Herrero and Arbeloa, 1989), and this delayed growth was due to a slow basipetal maturation of the pistil. In this species, the obturator did not only act as a gate to open the access to the ovule, but also seemed to close it since, following the secretory phase and pollen tube passage, callose was layered down on the obturator, preventing additional pollen tubes to pass by (Arbeloa and Herrero, 1987).

In other species with chalazogamy a different structure with a similar role has been described. In pistachio (*Pistacia vera)*, a chalazogamous species (Martinez-Palle and Herrero, 1998), concomitantly with the arrival of the pollen tubes to the base of the style, a protuberance (the ponticulus) develops in the uppermost area of the funiculus, filling the physical gap between the base of the style and the ovule (Martinez-Palle and Herrero, 1994). A ponticulus has also been described in other species of the Anacardiaceae such as mango (*Mangifera indica*) (Joel and Eisenstein, 1980; De-Wet et al., 1986; Bachelier and Endress, 2009). A similar situation has been described in Fagales, where, interestingly, in *Juglans* (Luza and Polito, 1991) both chalazogamy and porogamy -the entry of the pollen tube into the ovule through the micropyle- could be observed, depending on the developmental stage of the ovule. Chalazogamy has also been reported in hazelnut (*Corylus heterophylla*) that shows a delayed fertilization; in this case, the pollen tube grows through the chalaza 52 or 55 days after pollination and this could be related to the delayed development of the ovule whose ovule primordium was observed 20 days after blooming (Liu et al., 2014).

Sporophytic control of pollen tube growth is also observed in the integuments, which surround the nucellus that embeds the embryo sac forming the micropyle. Most angiosperms show bitegmic ovules compared to the single integument present in most extant and fossil gymnosperms (Herr, 1995; Endress, 2011; Lora et al., 2015). In evolutionarily derived angiosperms, the outer integument seems to be involved in providing cues for pollen tube guidance toward the embryo sac (Herrero, 2000, 2001, 2003). The role of the inner integument on pollen tube growth was also suggested by the *Arabidopsis pop2* mutant that shows higher concentration of GABA in the inner integument associated to reduced pollen tube guidance (Palanivelu et al., 2003). Moreover, in *Arabidopsis*, a truncated version of a protein disulfide isomerase PDIL2-1, expressed in the sporophytic tissue with higher expression in the micropyle of mature ovules, affect ovule structure, and impede embryo sac development disrupting pollen tube guidance (Wang et al., 2008). In the *Arabidopsis inner no outer* (*ino*) mutant lacking the outer integument, pollen tubes wander in the ovary and rarely reach the micropyle, although this could also be related to the absence of the embryo sac (Skinner and Gasser, 2009), since synergid cells play a role on pollen tube guidance (Higashiyama et al., 2001; Higashiyama and Hamamura, 2008; Kessler and Grossniklaus, 2011). However, the situation was different in a similar ovule *ino* mutant in *Annona squamosa* (magnoliids), which showed pollen tubes targeting the micropyle (Lora et al., 2011b). Further work is required to elucidate if this was due to the presence of an embryo sac in these ovules or to a late recruitment of the outer integument for pollen tube guidance in angiosperms, since in this species as in other *Annona* species the outer integument is retracted and does not embrace the micropyle.

To date, several molecules have emerged as major players involved in the cell–cell communications between synergids/egg apparatus and pollen tube (reviewed in Higashiyama and Takeuchi, 2015; Qu et al., 2015) supporting the main role of the female gametophyte in pollen tube reception (Kessler and Grossniklaus, 2011). Examples of those molecules include the defensin-like polypeptide LUREs (Okuda et al., 2009) in *Torenia*, the homolog AtLURE1 (Takeuchi and Higashiyama, 2012), the transcription factor MYB98 (Kasahara et al., 2005), the FERONIA (Escobar-Restrepo et al., 2007), and, recently, TURAN and EVA (Lindner et al., 2015) in *Arabidopsis*, or ZmEA1 that is expressed in the egg apparatus of maize (Marton et al., 2005). Recent studies have also shown the role of ROS on pollen tube growth (Potocký et al., 2007) and on the rupture of the pollen tube and subsequent fertilization in *Arabidopsis* (Duan et al., 2014),

Although less work has been done in gymnosperms, studies on the composition of the pollination drop has shown similar components to those of the stigma and style (Coulter et al., 2012), suggesting an involvement of these molecules on pollen–pistil interaction. Additional functional studies for these molecules to show their putative role in pollen tube guidance or pollen tube reception are needed. Work in angiosperms converges to the point of a sophisticated pollen–pistil interaction all along the different pistil territories the pollen tube has to traverse. Yet the question remains on the significance of this interaction and why along evolution the pollen tube journey seems to gain in complexity and difficulty. This complexity appears to share a common ground: a bigger control of the sporophyte.

## Toward a Major Control of the Sporophyte

When comparing gymnosperms, basal angiosperms and evolutionarily derived eudicots, evolution seems to have complicated what virtually could be simpler. The continuous adding of layers to envelop the embryo sac complicates pollen tube growth that in angiosperms has to often grow long distances within the sporophytic tissues of the flower. In angiosperms, closed carpels provide opportunities for an intense pollen–pistil interaction, whereas in gymnosperms the main interaction of pollen with the female sporophyte takes place at the micropyle in the pollination drop (**Figure 1**). Mulcahy (1979) proposed that the combination of closed carpels (syncarpy) and insect pollination could increase the chances for male gametophytic competition and selection enhancing the ability of natural selection to act on the gametophytic phase of the life cycle of angiosperms helping to understand the adaptive success of flowering plants.

The presence of physical barriers, such as dichogamy, monoecy, dioecy, or floral heteromorphy, between the female and male parts to avoid self-fertilization have developed in both gymnosperms and angiosperms (Zavada and Taylor, 1986). Moreover, the rejection of pollen from unrelated species, or incongruity (Hogenboom, 1975), has been documented in both gymnosperms (Mugnaini et al., 2007; Jin et al., 2012), and angiosperms (Heslop-Harrison, 2000). It is difficult to understand how minute secretions allow discerning between conspecific and heterospecific pollen, since the basic ingredients of artificial pollen germination media are very similar for the different species. This discernment suggests a precise male–female cross talk, right from the first encounter (McInnis et al., 2006).

More intriguing is the possible pollen selection in intraspecific matings. The most common way to avoid self-fertilization in angiosperms is self-incompatibility (SI) (de Nettancourt, 2001; Iwano and Takayama, 2012; Sawada et al., 2014). Although evidence of failure of pollen tube growth before fertilization has been reported in conifers (Kormutak, 1999; Takaso et al., 1996; Gelbart and von Aderkas, 2002), this is still a subject of debate (Little et al., 2014), and, in a good number of instances, selection against self-fertilization in gymnosperms appears to be related to post-fertilization events (Orr-Ewing, 1957; Hagman and Mikkola, 1963; Mergen et al., 1965; Williams et al., 2001; Owens et al., 2005).

In angiosperms, the evidence of SI is widespread, but the very different types of incompatibility recorded in different families suggest that SI has arisen *de novo* several times in independent lineages of flowering plants (Ferrer and Good, 2012; Gibbs, 2014). It is estimated that half of angiosperm species show SI (Dresselhaus and Franklin-Tong, 2013; Gibbs, 2014), but only in a few families has SI been characterized in detail. Three main types of SI have been described, sporophytic self-incompatibility (SSI), gametophytic self-incompatibility (GSI), and late acting-self incompatibility (LSI). SSI has only been reported in six families (Gibbs, 2014) and seems to be restricted to eudicots (Allen and Hiscock, 2008); GSI has been reported in species of 18 families (Gibbs, 2014) and is the most abundant SI mechanism in flowering plants, present in all major clades of monocots and eudicots (Allen and Hiscock, 2008). Excellent recent reviews have addressed the evolution, diversity and molecular mechanisms of the different self-incompatibility systems (de Nettancourt, 2001; Takayama and Isogai, 2005; Franklin-Tong, 2008; Iwano and Takayama, 2012; Gibbs, 2014).

Different empirical evidence shows a high overlap in the genes expressed during the gametophytic and the sporophytic phases of the angiosperm life cycle (Ottaviano and Mulcahy, 1989; Mascarenhas, 1990; Honys and Twell, 2003) as well as a correlation between higher pollen competition and selection and traits in the following sporophytic generation (Hormaza and Herrero, 1992, 1994). Pollen selection is highly dependent on pollen competition, and the pistil seems to be well devised to favor competition among growing male gametophytes (Hormaza and Herrero, 1992, 1994, 1996b; Herrero and Hormaza, 1996), starting by the promotion of an accumulation of a high number of pollen grains in the stigma. This allows the style to act as a long sieve, with a progressive reduction in both nutritive resources and space, that finally results in a reduced number of pollen tubes able to reach the ovary (Herrero, 1992). However, this situation appears to be different between the ANITA grade, magnoliids and eudicots. The stigmas of the ANITA grade and magnoliids show extragynoecial compitum that allows pollen tube growth between the stigmas of the different carpels (Endress and Igersheim, 2000; Williams et al., 2010; Lora et al., 2011a). Moreover, the carpels of he ANITA grade and magnoliids are generally separate (apocarpous) and show a short style (Orban and Bouharmont, 1995; Pontieri and Sage, 1999; Lora et al., 2010; Galati et al., 2016). As a consequence, the main competition among male gametophytes occurs in this extragynoecial compitum. In fact, extragynoecial pollen tube growth is widespread in apocarpous species (Wang et al., 2012). In contrast, most evolutionarily derived angiosperms (83%) show fusion of the carpels forming a syncarpous gynoecium (Endress, 1982). The syncarpous gynoecium mostly has an intragynoecial compitum that provides more protection from adverse environmental conditions, and allows a more intense pollen tube competition and selection (Endress, 1982). This increased pollen competition is reinforced in evolutionarily derived angiosperms by the common presence of long styles that act as long sieves reducing basipetally both the space and the nutrients available for growing pollen tubes (Herrero and Hormaza, 1996; Distefano et al., 2011).

In theory, this gametophytic competition and selection should not be restricted to the male gametophytes but it is more difficult to prove in the female gametophytes. Nevertheless, recently, Bachelier and Friedman (2011) have shown the occurrence of gametophytic competition among female gametophytes within the single ovule in the early-divergent angiosperm *Trimenia moorei.* It will be of interest to analyze if this could be the case in other angiosperm lineages although, in any case, the likelihood of a significant selection pressure will always be higher in the male gametophytes due to their higher population number.

Although the presence of pollen competition in plants has been shown in a high number of cases (Hormaza and Herrero, 1994), it is still difficult to ascertain whether this competition finally results in selection for particular traits or adaptation to particular conditions that could result in a change in the gene frequencies of the next sporophytic generation (Baskin and Baskin, 2015). Evidence has been obtained mainly in two areas. Pollen behavior in response to different environmental factors has been shown to differ among different genotypes (Hormaza and Herrero, 1992; Hedhly et al., 2005a), and plants exposed to a selection pressure during the reproductive process may produce an offspring more adapted to these conditions than when the same crosses are performed under controlled conditions (Hormaza and Herrero, 1996a; Hedhly et al., 2009; Hedhly, 2011). Recent work using molecular approaches provides additional evidence that genome evolution could be affected by pollen competition. Thus, Arunkumar et al. (2013) showed in *Capsella grandiflora* that selection had more detectable effects on pollen-exclusive genes than on seedling-exclusive genes whereas Chettoor et al. (2014) observed that selective pressures based on the male gametophytic function result in high effects on the maize plant genome. Additional work assessing paternity of the offspring shows that pollen tube competition could also result in sexual selection in plants (Hedhly et al., 2015).

All this converges to the point that the pistil exerts a dual support/constrain strategy that may result in gametophyte competition and selection (Herrero and Hormaza, 1996). Thus the sporophyte supports pollen tube heterotrophic growth, but also promotes pollen competition and selection with spatial and nutritive constraints and with self-incompatibility systems that involve cell-cell recognition. In fact, if gametophytic selection is mainly based in the growth rate of pollen tubes, we would expect that the traits responsible for rapid pollen tube growth should be rapidly fixed in the populations. Sporophytic control would result in the fact that the best male–female combinations are favored providing an advantage to the best suited male gametophytes for a given female genotype which could explain why genetic variation for male gametophytic fitness has been maintained in plant populations (Herrero and Hormaza, 1996; Hormaza and Herrero, 1996a). In angiosperms the development of the carpel, while providing further protection for the female gametophyte and the seed, also gives an enhanced opportunity for male–female cross talk. This results first in a change in the meeting place from the pollination drop of gymnosperms to the secretory stigma of angiosperms. But, more interestingly, it also results in changes in the landscape that the pollen tube has to traverse to reach the megagametophyte. The style provides ample opportunity for pollen competition and selection, and the ovary shows a close control of pollen tube access to the ovule. This change in the territory that the pollen tube has to traverse along its journey results in a process more protected from external environmental factors, but also in the sporophyte gaining control over the gametophyte. As a result, the interaction between the gametophyte and the sporophytic tissues of the flower seems to be an arena with implications for plant diversity and evolution.

## Author Contributions

JL, JH, and MH wrote and reviewed the final version of the manuscript.

## Conflict of Interest Statement

The authors declare that the research was conducted in the absence of any commercial or financial relationships that could be construed as a potential conflict of interest.
